# Supplementary material for: Early use of barbiturates is associated with increased mortality in traumatic brain injury patients from a propensity score-based analysis of a prospective cohort
Source: PLoS One. 2022 May 4;17(5):e0268013. doi: 10.1371/journal.pone.0268013 (PMC9067881; doi:10.1371/journal.pone.0268013)

**Early use of barbiturates is associated with increased mortality in traumatic brain injury patients from a propensity score-based analysis of a prospective cohort.**

Maxime Léger, MD ^1,2*^, [mxmleger@gmail.com](mailto:mxmleger@gmail.com)

Denis Frasca, MD,PhD ^2,3^, [denis.frasca@gmail.com](mailto:denis.frasca@gmail.com)

Antoine Roquilly, MD,PhD ^4^, [antoine.roquilly@chu-nantes.fr](mailto:antoine.roquilly@chu-nantes.fr)

Philippe Seguin, MD,PhD ^5^, [philippe.seguin@chu-rennes.fr](mailto:philippe.seguin@chu-rennes.fr)

Raphaël Cinotti, MD, PhD ^4^, [raphael.cinotti@chu-nantes.fr](mailto:raphael.cinotti@chu-nantes.fr)

Claire Dahyot-Fizelier, MD, PhD ^3^, [claire.dahyot-fizelier@chu-poitiers.fr](mailto:claire.dahyot-fizelier@chu-poitiers.fr)

Karim Asehnoune, MD, PhD ^4^, [Karim.ASEHNOUNE@chu-nantes.fr](mailto:Karim.ASEHNOUNE@chu-nantes.fr)

Florent Le Borgne, PhD ^2,6^, [fleborgne@idbc.fr](mailto:fleborgne@idbc.fr)

Thomas Gaillard, MD ^1^, ThGaillard@chu-angers.fr

Yohann Foucher, PhD ^2,7^, [yohann.foucher@univ-nantes.fr](mailto:yohann.foucher@univ-nantes.fr)

Sigismond Lasocki, MD, PhD ^1^, sigismond@lasocki.com

for AtlanREA group, [atlanreagroup@gmail.com](mailto:atlanreagroup@gmail.com)

^1^ Département d’Anesthésie Réanimation, Centre Hospitalier Universitaire d’Angers, Angers, France.

^2^ INSERM UMR 1246 - SPHERE, Nantes University, Tours University, Nantes, France.

^3^ Département d’Anesthésie Réanimation, Centre Hospitalier Universitaire de Poitiers, Poitiers, France.

^4^ Département d’Anesthésie Réanimation, Centre Hospitalier Universitaire de Nantes, Nantes, France.

^5^ Département d’Anesthésie Réanimation, Centre Hospitalier Universitaire de Rennes, Rennes, France.

^6^ IDBC-A2COM, Pacé, France.

^7^ Centre Hospitalier Universitaire de Nantes, Nantes, France.

**Corresponding author :** Dr Maxime Léger – Département d’Anesthésie Réanimation, CHU d’Angers, 4 rue Larrey 49100 Angers, France – Tel: +33 241353635 – Fax: +33 241353967 – [mxmleger@gmail.com](mailto:mxmleger@gmail.com)

Twitter: @mxmleger

**Supplemental Digital Content**

**Table S1.** Results of the multivariable logistic models leading to the propensity score.

To take into account possible confounding variables, we used the method of weighting according to inverse probability (Inverse Probability Weighting - IPW) of the Propensity Score (PS). The PS was estimated by a multivariable logistic regression with splines on continuous covariates to ensure the log-linearity assumption. Stabilized weights have been used in order to obtain a pseudo dataset with similar sample size than the original one and to estimate the average effect of the exposure (Average Treatment effect in the Entire population - ATE). To provide a relative measure of the effect, a logistic regression was estimated maximizing the weighted likelihood and using a robust estimator for the variance. The logistic regression leading to the propensity score is presented on table S2. Influential values were detected by a Cook distance greater than 1 in absolute value. Model was adjusted on center (effect was included in the propensity score).

For each outcome, the variables integrated into the model were those significantly associated with both the treatment (initiation of barbiturates) and the analyzed outcome. The significance threshold was defined in univariate analysis with a p-value < 0.2.

**Table S1.a.** Results of the multivariable logistic models leading to the propensity score regarding the survival analysis in ICU (n= 300).

|  | **OR** | **95% CI** | **p-value** |
| --- | --- | --- | --- |
| **Osmotherapy** | 0.43 | [0.22 ; 0.84] | 0.0128 |
| **SAP ≤ 90 mmHg before admission** | 0.87 | [0.45 ; 1.67] | 0.6764 |
| **Evacuation of subdural or extradural hematoma** | 0.64 | [0.34 ; 1.20] | 0.1621 |
| **Blood transfusion before admission** | 0.42 | [0.21 ; 0.86] | 0.0166 |
| **Arterial pH** | 1.09 | [0.59 ; 2.02] | 0.7872 |
| **Serum proteins (g/L)** | 1.07 | [0.53 ; 2.15] | 0.8572 |
| **Serum glucose (mmol/l)** | 1.20 | [0.67 ; 2.17] | 0.5384 |
| **Serum calcium (mmol/l)** | 0.68 | [0.35 ; 1.31] | 0.2518 |

CI, Confidence Interval; OR, Odds Ratio; SAP, Systolic Arterial Pressure

**Table S1.b.** Results of the second multivariable logistic models leading to the propensity score regarding the survival analysis in ICU (n= 279).

|  | **OR** | **95% CI** | **p-value** |
| --- | --- | --- | --- |
| **Osmotherapy** | 0.46 | [0.23 ; 0.93] | 0.0310 |
| **Blood transfusion before admission** | 0.49 | [0.23 ; 1.05] | 0.0680 |
| **Serum glucose ≥ 8 mmol/l** | 1.09 | [0.57 ; 2.08] | 0.7850 |
| **IMPACT TBI score ≥ 9** | 0.84 | [0.44 ; 1.58] | 0.5860 |
| **Arterial pH** |  |  | 0.8631 |
| **spline - 1** | 2.86 | [0.16 ; 50.10] |  |
| **spline - 2** | 1.07 | [0.13 ; 9.16] |  |
| **spline - 3** | 3.03 | [0.01 ; 1471.37] |  |
| **spline - 4** | 1.62 | [0.10 ; 25.41] |  |
| **Serum proteins (g/L)** |  |  | 0.9461 |
| **spline - 1** | 1.54 | [0.14 ; 17.13] |  |
| **spline - 2** | 1.74 | [0.12 ; 25.66] |  |
| **spline - 3** | 15.47 | [0.01 ; 26536.32] |  |
| **spline - 4** | 12.67 | [0.00 ; 1148159.12] |  |
| **Serum calcium (mmol/L)** |  |  | 0.2573 |
| **spline - 1** | 0.00 | [0.00 ; 0.85] |  |
| **spline - 2** | 0.00 | [0.00 ; 2.14] |  |
| **spline - 3** | 0.00 | [0.00 ; 0.74] |  |
| **spline - 4** | 0.03 | [0.00 ; 2682.15] |  |
| CI, confidence interval; OR, odds ratio. | | | |

**Table S1.c.** Results of the multivariable logistic model leading to the propensity score regarding the analysis of the occurrence of ventilator-associated pneumonia (n= 372).

|  | **OR** | **95% CI** | **p-value** |
| --- | --- | --- | --- |
| **Decompressive craniectomy** | 0.74 | [0.40 ; 1.36] | 0.3292 |
| **Age (years)** |  |  | 0.1658 |
| **spline - 1** | 0.34 | [0.02 ; 5.32] |  |
| **spline - 2** | 2.39 | [0.19 ; 29.71] |  |
| **spline - 3** | 2.12 | [0.15 ; 29.25] |  |
| **Serum Creatinine (mmol/L)** | 1.39 | [0.84 ; 2.31] | 0.2009 |
| CI, Confidence Interval; OR, Odds Ratio. | | | |

**Table S1.d.** Results of the multivariable logistic model leading to the propensity score regarding the analysis of the occurrence of dichotomized GOS (n= 251).

|  | **OR** | **95% CI** | **p-value** |
| --- | --- | --- | --- |
| **Osmotherapy** | 0.35 | [0.17 ; 0.71] | 0.0038 |
| **Blood transfusion before admission** | 0.57 | [0.28 ; 1.17] | 0.1269 |
| **Platelets (count/mm^3^)** |  |  | 0.7933 |
| **spline - 1** | 0.37 | [0.00 ; 72.16] |  |
| **spline - 2** | 3.61 | [0.20 ; 64.14] |  |
| **spline - 3** | 0.26 | [0.01 ; 10.88] |  |
| **Arterial pH** |  |  | 0.7095 |
| **spline - 1** | 1.28 | [0.00 ; 649.00] |  |
| **spline - 2** | 2.10 | [0.07 ; 58.96] |  |
| **spline - 3** | 0.49 | [0.01 ; 20.87] |  |
| **Serum calcium (mmol/L)** | 0.64 | [0.34 ; 1.22] | 0.1780 |
| CI, confidence interval; OR, odds ratio. | | | |

**Table S1.e.** Results of the second multivariable logistic models leading to the propensity score regarding the analysis of the occurrence of dichotomized GOS (n= 230).

|  | **OR** | **95% CI** | **p-value** |
| --- | --- | --- | --- |
| **Osmotherapy** | 0.34 | [0.16 ; 0.71] | 0.0042 |
| **Blood transfusion before admission** | 0.45 | [0.21 ; 0.98] | 0.0440 |
| **Arterial pH ≥7.3** | 0.58 | [0.29 ; 1.16] | 0.1255 |
| **Serum calcium ≥1.9 mmol/L** | 2.17 | [1.08 ; 4.37] | 0.0295 |
| **IMPACT TBI score ≥10** | 0.97 | [0.50 ; 1.86] | 0.9189 |
| **Platelets (count/mm^3^)** |  |  | 0.3807 |
| **spline - 1** | 0.13 | [0.00 ; 4.51] |  |
| **spline - 2** | 0.17 | [0.01 ; 2.40] |  |
| **spline - 3** | 0.00 | [0.00 ; 3.58] |  |
| **spline - 4** | 0.24 | [0.01 ; 4.29] |  |
| CI, confidence interval; OR, odds ratio. | | | |

**Table S1.f.** Results of the multivariable logistic models leading to the propensity score regarding the survival analysis in ICU for the sensitivity analysis: all patients who were treated with barbiturates at any time during their stay versus the control group (n= 279).

|  | **OR** | **95% CI** | **p-value** |
| --- | --- | --- | --- |
| **Osmotherapy** | 0.53 | [0.30 ; 0.91] | 0.0220 |
| **SAP ≤ 90 mmHg before admission** | 0.78 | [0.43 ; 1.42] | 0.4199 |
| **Blood transfusion before admission** | 0.87 | [0.46 ; 1.62] | 0.6517 |
| **Plasma bicarbonate ≥21 mmol/L** | 1.01 | [0.60 ; 1.71] | 0.9620 |
| **Fibrinogen (g/L)** |  |  | 0.2506 |
| **spline - 1** | 1.20 | [0.25 ; 5.75] |  |
| **spline - 2** | 6.15 | [0.96 ; 39.56] |  |
| **spline - 3** | 1.39 | [0.03 ; 58.77] |  |
| **spline - 4** | 0.49 | [0.01 ; 16.78] |  |
| **Serum calcium (mmol/L)** |  |  | 0.3839 |
| **spline - 1** | 0.28 | [0.01 ; 6.23] |  |
| **spline - 2** | 0.85 | [0.07 ; 10.66] |  |
| **spline - 3** | 0.55 | [0.00 ; 909.91] |  |
| **spline - 4** | 22.83 | [0.01 ; 43742.44] |  |
| **ISS score** |  |  | 0.9142 |
| **spline - 1** | 0.77 | [0.27 ; 2.23] |  |
| **spline - 2** | 1.29 | [0.32 ; 5.17] |  |
| **spline - 3** | 0.86 | [0.08 ; 8.87] |  |
| **spline - 4** | 0.36 | [0.04 ; 3.12] |  |
| CI, confidence interval; ICU, OR, odds ratio, SAP, Systolic Arterial Pressure | | | |

**Table S2.** The PS-adjusted samples.

**Table S2.a.** The PS-adjusted sample for the survival analysis (n= 302, weighted sample).

|  | **Whole sample** | | **Barbiturates group** | | **Control group** | | **Standardized** |
| --- | --- | --- | --- | --- | --- | --- | --- |
|  | **n** | **%** | **n** | **%** | **n** | **%** | **differences (%)** |
| **Osmotherapy** | 197.6 | 65.3 | 46.5 | 62.2 | 151.1 | 66.3 | 8.6 |
| **SAP ≤ 90 mmHg before admission** | 79.1 | 26.2 | 17.3 | 23.1 | 61.9 | 27.2 | 9.4 |
| **Evacuation of subdural or extradural hematoma** | 77.0 | 25.4 | 16.6 | 22.2 | 60.4 | 26.5 | 10.0 |
| **Blood transfusion before admission** | 85.7 | 28.3 | 20.3 | 27.1 | 65.4 | 28.7 | 3.6 |
|  | **m** | **SD** | **m** | **SD** | **m** | **SD** |  |
| **Arterial pH** | 7.3 | 0.1 | 7.3 | 0.1 | 7.3 | 0.1 | 11.6 |
| **Serum proteins (g/L)** | 53.7 | 10.9 | 52.8 | 9.3 | 54.0 | 11.4 | 11.1 |
| **Serum glucose (mmol/L)** | 8.6 | 3.0 | 8.9 | 3.5 | 8.4 | 2.8 | 15.4 |
| **Serum calcium (mmol/L)** | 2.0 | 0.2 | 2.0 | 0.2 | 2.0 | 0.2 | 5.0 |

m, weighted mean; SAP, Systolic Arterial Pressure; sd, weighted standard deviation;

**Table S2.b.** The PS-adjusted sample for the survival analysis with the second model (TBI IMPACT score)

(n= 278, weighted sample).

|  | **Whole sample** | | **Barbiturates group** | | **Control group** | | **Standardized** |
| --- | --- | --- | --- | --- | --- | --- | --- |
|  | **n** | **%** | **n** | **%** | **n** | **%** | **differences (%)** |
| **Osmotherapy** | 184.3 | 66.4 | 43.5 | 69.7 | 140.8 | 65.4 | 9.2 |
| **Blood transfusion before admission** | 77.8 | 28.0 | 16.1 | 25.9 | 61.7 | 28.7 | 6.3 |
| **Serum glucose ≥8 mmol/l** | 156.1 | 56.2 | 37.4 | 59.9 | 118.7 | 55.1 | 9.7 |
| **IMPACT TBI score ≥9** | 137.2 | 49.4 | 28.8 | 46.2 | 108.4 | 50.4 | 8.4 |
|  | **m** | **SD** | **m** | **SD** | **m** | **SD** |  |
| **Arterial pH** | 7.3 | 0.1 | 7.3 | 0.1 | 7.3 | 0.1 | 11.8 |
| **Serum proteins (g/L)** | 53.7 | 10.9 | 53.2 | 10.0 | 53.8 | 11.2 | 6.4 |
| **Serum calcium (mmol/L)** | 2.0 | 0.2 | 2.0 | 0.2 | 2.0 | 0.2 | 5.4 |
| m, mean; SD, standard deviation. | | | | | | | |

**Table S2.c.** The PS-adjusted sample for analysis of VAP occurrences (n= 374; weighted sample).

|  | **Whole sample** | | **Barbiturates group** | | **Control group** | | **Standardized** |
| --- | --- | --- | --- | --- | --- | --- | --- |
|  | **n** | **%** | **n** | **%** | **n** | **%** | **differences (%)** |
| **Decompressive craniectomy** | 70.3 | 18.8 | 16.9 | 17.5 | 53.4 | 19.3 | 4.7 |
|  | **m** | **SD** | **m** | **SD** | **m** | **SD** |  |
| **Age (years)** | 40.6 | 18.9 | 40.8 | 19.2 | 40.5 | 18.8 | 1.6 |
| **Serum Creatinine (mmol/L)** | 77.7 | 30.6 | 81.1 | 32.6 | 76.6 | 29.8 | 14.7 |
| m, weighted mean; SD, standard deviation. | | | | | | | |

**Table S2.d.** The PS-adjusted sample for analysis of dichotomized Glasgow Outcome Scale score at 3 months (n= 250; weighted sample).

|  | **Whole sample** | | **Barbiturates group** | | **Control group** | | **Standardized** |
| --- | --- | --- | --- | --- | --- | --- | --- |
|  | **n** | **%** | **n** | **%** | **n** | **%** | **differences (%)** |
| **Osmotherapy** | 169.0 | 67.7 | 43.6 | 70.4 | 125.3 | 66.8 | 7.7 |
| **Blood Transfusion before admission** | 77.7 | 31.1 | 20.3 | 32.8 | 57.3 | 30.6 | 4.8 |
|  | **m** | **SD** | **m** | **SD** | **m** | **SD** |  |
| **Platelets (count/mm^3^)** | 175.3 | 72.3 | 172.2 | 73.4 | 176.3 | 72.1 | 5.6 |
| **Arterial pH** | 7.3 | 0.1 | 7.3 | 0.1 | 7.3 | 0.1 | 3.1 |
| **Serum calcium (mmol/L)** | 1.9 | 0.2 | 1.9 | 0.2 | 2.0 | 0.2 | 14.5 |
| m, weighted mean; SD, standard deviation. | | | | | | | |

**Table S2.e.** The PS-adjusted sample for analysis of dichotomized Glasgow Outcome Scale score at 3 months with the second model (TBI IMPACT score) (n= 230; weighted sample).

|  | **Whole sample** | | **Barbiturates group** | | **Control group** | | **Standardized** |
| --- | --- | --- | --- | --- | --- | --- | --- |
|  | **n** | **%** | **n** | **%** | **n** | **%** | **differences (%)** |
| **Osmotherapy** | 151.9 | 66.0 | 38.2 | 65.4 | 113.6 | 66.3 | 1.9 |
| **Blood transfusion before admission** | 70.9 | 30.8 | 18.3 | 31.3 | 52.6 | 30.7 | 1.4 |
| **Arterial pH ≥7.3** | 113.7 | 49.4 | 27.3 | 46.6 | 86.4 | 50.4 | 7.4 |
| **Serum calcium ≥1.9 mmol/L** | 136.3 | 59.3 | 33.5 | 57.2 | 102.8 | 60.0 | 5.6 |
| **IMPACT TBI score ≥10** | 108.0 | 46.9 | 28.9 | 49.5 | 79.0 | 46.1 | 6.8 |
|  | **m** | **SD** | **m** | **SD** | **m** | **SD** |  |
| **Platelets (count/mm^3^)** | 176.1 | 70.4 | 173.3 | 69.0 | 177.0 | 71.2 | 5.3 |
| m, mean; SD, standard deviation. | | | | | | | |

**Table S2.f.** The PS-adjusted sample for the survival analysis of the sensitivity analysis: all patients who were treated with barbiturates at any time during their stay versus the control group (n= 279; weighted sample).

|  | **Whole sample** | | **Barbiturates group** | | **Control group** | | **Standardized** |
| --- | --- | --- | --- | --- | --- | --- | --- |
|  | **n** | **%** | **n** | **%** | **n** | **%** | **differences (%)** |
| **Osmotherapy** | 187.6 | 67.4 | 82.1 | 67.5 | 105.5 | 67.3 | 0.4 |
| **SAP ≤ 90 mmHg before admission** | 78.3 | 28.1 | 33.6 | 27.6 | 44.7 | 28.5 | 1.9 |
| **Blood transfusion before admission** | 88.7 | 31.9 | 39.0 | 32.0 | 49.8 | 31.7 | 0.7 |
| **Plasma bicarbonate ≥21 mmol/L** | 139.0 | 49.9 | 60.6 | 49.8 | 78.4 | 50.0 | 0.3 |
|  | **m** | **SD** | **m** | **SD** | **m** | **SD** |  |
| **Fibrinogen (g/L)** | 2.2 | 1.1 | 2.2 | 1.1 | 2.2 | 1.1 | 1.7 |
| **Serum calcium (mmol/L)** | 2.0 | 0.2 | 2.0 | 0.2 | 2.0 | 0.2 | 0.7 |
| **ISS score** | 29.4 | 14.7 | 29.5 | 14.8 | 29.3 | 14.7 | 1.8 |
| m, mean; SAP, Systolic Arterial Pressure ; SD, standard deviation. | | | | | | | |

**Table S3.** Description of the analyzed patients for the dichotomized Glasgow outcome score at 90 days versus excluded patients for missing data.

|  | **Overall (n=383)** | | | **Analyzed patients for dichotomized Glasgow outcome score at 90 days (n=283)** | | | **Excluded patients (n=100)** | | | **p-value** |
| --- | --- | --- | --- | --- | --- | --- | --- | --- | --- | --- |
|  | **NA** | **n** | **%** | **NA** | **n** | **%** | **NA** | **n** | **%** |  |
| **Center** | 0 |  |  | 0 |  |  | 0 |  |  | 0.0012 |
| **A** |  | 87 | 22.7 |  | 69 | 24.4 |  | 18 | 18.0 |  |
| **B** |  | 171 | 44.6 |  | 135 | 47.7 |  | 36 | 36.0 |  |
| **C** |  | 69 | 18.0 |  | 49 | 17.3 |  | 20 | 20.0 |  |
| **D** |  | 56 | 14.7 |  | 30 | 10.6 |  | 26 | 26.0 |  |
| **Male patient** | 0 | 308 | 80.4 | 0 | 225 | 79.5 | 0 | 83 | 83.0 | 0.4490 |
| **History of diabetes** | 5 | 22 | 5.8 | 2 | 16 | 5.7 | 3 | 6 | 6.2 | 0.0649 |
| **Chronic alcoholism** | 31 | 64 | 18.2 | 45 | 68 | 14.5 | 11 | 30 | 21.1 | 0.0608 |
| **Active smoking status** | 61 | 94 | 29.2 | 49 | 66 | 28.2 | 12 | 28 | 31.8 | 0.5251 |
| **SAP ≤ 90 mmHg before admission** | 7 | 105 | 27.9 | 5 | 82 | 29.5 | 2 | 23 | 23.5 | 0.2528 |
| **Hypoxia** | 10 | 279 | 74.8 | 6 | 211 | 76.2 | 4 | 68 | 70.8 | 0.3670 |
| **Blood transfusion before admission** | 1 | 99 | 25.9 | 0 | 80 | 28.3 | 1 | 19 | 19.2 | 0.0761 |
| **Glasgow score < 8** | 5 | 104 | 27.2 | 3 | 70 | 24.7 | 2 | 34 | 34.0 | 0.0909 |
| **Glasgow motor score** | 20 |  |  | 11 |  |  | 9 |  |  | 0.0814 |
| 6 |  | 35 | 9.6 |  | 28 | 10.3 |  | 7 | 7.7 |  |
| 5 |  | 63 | 17.4 |  | 51 | 18.8 |  | 12 | 13.2 |  |
| 4 |  | 82 | 22.6 |  | 53 | 19.5 |  | 29 | 31.9 |  |
| 3 |  | 36 | 9.9 |  | 29 | 10.6 |  | 7 | 7.7 |  |
| 2 |  | 34 | 9.4 |  | 24 | 8.8 |  | 10 | 11.0 |  |
| 1 |  | 113 | 31.1 |  | 87 | 32.0 |  | 26 | 28.6 |  |
| **Unreactive mydriasis or anisocoria** | 5 | 140 | 36.5 | 2 | 114 | 40.1 | 3 | 26 | 26.0 | 0.0122 |
| **CT scan classification**  **Marshall classification**  Diffuse injury I  Diffuse injury II  Diffuse injury III  Diffuse injury IV  Evacuated mass lesion V  Non-evacuated mass lesion VI | 0 | 10  89  33  25  151  75 | 2.6  23.2  8.6  6.5  39.5  19.6 | 0 | 5  64  24  22  107  61 | 1.8  22.6  8.5  7.8  37.8  21.5 | 0 | 5  25  9  3  44  14 | 5.0  25.0  9.0  3.0  44.0  14.0 | 0.1255 |
| **Osmotherapy at admission** | 2 | 255 | 66.9 | 2 | 188 | 66.9 | 0 | 67 | 67.0 | 0.9860 |
| **Evacuation of subdural or extradural hematoma at admission** | 0 | 108 | 28.2 | 0 | 72 | 25.4 | 0 | 36 | 36.0 | 0.0437 |
| **External ventricular drain at admission** | 0 | 28 | 7.3 | 0 | 23 | 8.1 | 0 | 5 | 5.0 | 0.3018 |
| **Evacuation of cerebral hematoma or lobectomy at admission** | 0 | 17 | 4.4 | 0 | 8 | 2.8 | 0 | 9 | 9.0 | 0.0196 |
| **Decompressive craniectomy at admission** | 0 | 74 | 19.3 | 0 | 53 | 18.7 | 0 | 21 | 21.0 | 0.6208 |
|  | **NA** | **m** | **sd** | **NA** | **m** | **sd** | **NA** | **m** | **sd** |  |
| **Age (years)** | 0 | 40.5 | 18.7 | 0 | 41.1 | 19.4 | 0 | 38.9 | 16.6 | 0.2673 |
| **BMI (kg.m-2)** | 48 | 24.3 | 4.5 | 39 | 24.4 | 4.6 | 9 | 23.8 | 4.5 | 0.2710 |
| **Intracranial pressure at admission (mm Hg)** | 17 | 22.0 | 15.8 | 14 | 22.8 | 15.7 | 3 | 19.7 | 16.0 | 0.0974 |
| **Hemoglobin (g/dL)** | 3 | 11.0 | 2.5 | 1 | 11.0 | 2.5 | 2 | 11.2 | 2.3 | 0.4672 |
| **Leukocytes (count/mm^3^)** | 4 | 17.9 | 7.1 | 1 | 18.2 | 7.3 | 3 | 16.9 | 6.3 | 0.0967 |
| **Prothrombin (%)** | 15 | 69.6 | 18.9 | 9 | 68.3 | 19.2 | 6 | 73.4 | 17.8 | 0.0191 |
| **Platelets (count/mm^3^)** | 4 | 178.4 | 70.0 | 1 | 177.1 | 70.4 | 3 | 182.3 | 69.2 | 0.0232 |
| **Fibrinogen (g/L)** | 70 | 2.3 | 1.2 | 38 | 2.2 | 1.1 | 32 | 2.7 | 1.6 | 0.0232 |
| **Arterial lactate (mmol/L)** | 52 | 2.8 | 2.3 | 32 | 2.9 | 2.5 | 20 | 2.4 | 1.4 | 0.0079 |
| **Arterial pH** | 4 | 7.3 | 0.1 | 2 | 7.3 | 0.1 | 2 | 7.3 | 0.1 | 0.0005 |
| **Bicarbonate (mmol/L)** | 4 | 20.9 | 4.1 | 3 | 20.4 | 4.0 | 1 | 22.2 | 4.3 | 0.0005 |
| **PaO2/FiO2 ratio** | 14 | 321.0 | 179.4 | 8 | 313.3 | 179.2 | 6 | 343.5 | 179.0 | 0.0069 |
| **Serum creatinine (mmol/L)** | 4 | 78.5 | 31.4 | 1 | 80.7 | 33.3 | 3 | 72.2 | 23.9 | 0.0069 |
| **Serum proteins (g/L)** | 9 | 54.3 | 11.0 | 4 | 53.4 | 11.3 | 5 | 57.1 | 9.6 | 0.0022 |
| **Serum glucose (mmol/L)** | 31 | 8.7 | 3.4 | 17 | 9.0 | 3.6 | 14 | 8.0 | 2.8 | 0.0105 |
| **Serum urea (mmol/L)** | 9 | 4.9 | 2.1 | 5 | 5.0 | 2.2 | 4 | 4.5 | 1.7 | 0.0215 |
| **Serum calcium (mmol/L)** | 42 | 2.0 | 0.2 | 27 | 1.9 | 0.2 | 15 | 2.0 | 0.2 | 0.0180 |
| **SAPS II score** | 19 | 46.0 | 12.4 | 12 | 47.3 | 12.2 | 7 | 42.2 | 12.2 | 0.0006 |
| **ISS score** | 2 | 27.1 | 14.7 | 2 | 27.5 | 15.4 | 0 | 25.9 | 12.5 | 0.2953 |
| **IMPACT TBI score** | 38 | 8.8 | 4.3 | 23 | 9.1 | 4.3 | 15 | 7.6 | 4.1 | 0.0041 |

p-values are obtained using Chi-square test for categorical variables and using Student t-test for continuous variables.

BMI, Body Mass Index; FIO2, Fraction of Inspired Oxygen; GCS, Glasgow Coma Score; ISS, Injury Severity Score; NA, Not Available; PaO2, arterial partial Pressure of Oxygen; SAP, Systolic Arterial Pressure; SD, standard deviation; SAPS, Simplified Acute Physiology Score.

**Figure S1**. Propensity score distributions

**Figure S1.a.** Propensity score distribution for survival analysis according to the exposure groups of interest.


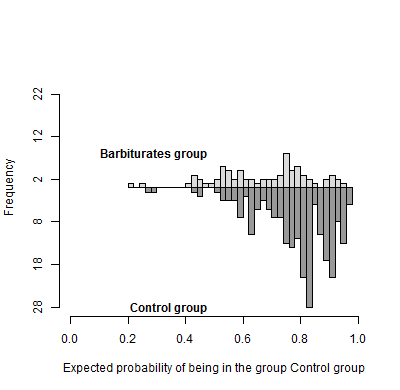


**Figure S1.b.** Propensity score distribution for survival analysis with the second model (TBI IMPACT score) according to the exposure groups of interest.


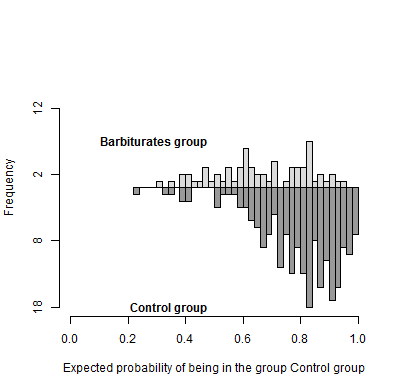


**Figure S1.c.** Propensity score distribution for incidence analysis of ventilator-associated pneumonia according to the exposure groups of interest.


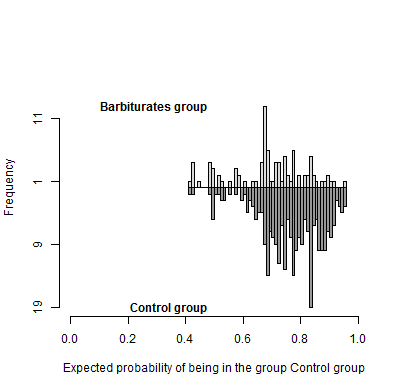


**Figure S1.d.** Propensity score distribution for analysis of dichotomized Glasgow Outcome Scale score at 3 months according to the exposure groups of interest.


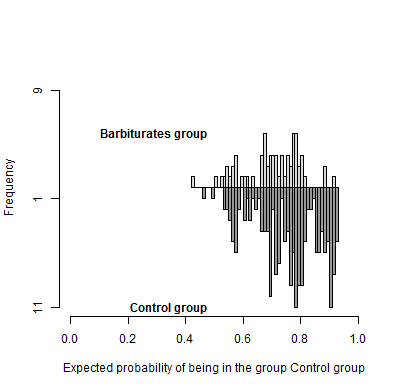


**Figure S1.e.** Propensity score distribution for analysis of dichotomized Glasgow Outcome Scale score at 3 months with the second model (TBI IMPACT score) according to the exposure groups of interest.


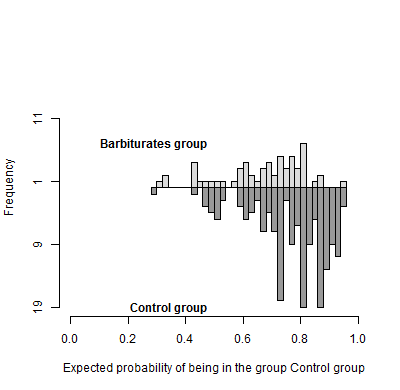


**Figure S1.f.** Propensity score distribution for survival analysis according to the exposure groups of interest, for the sensitivity analysis: all patients who were treated with barbiturates at any time during their stay versus the control group.


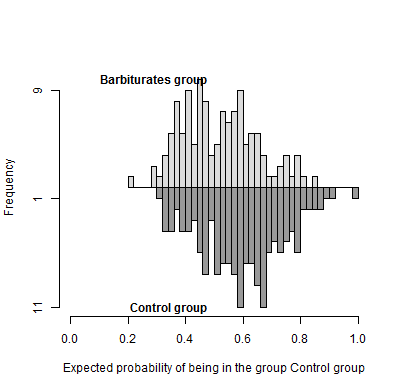

Supplement: S1 File — (DOCX) [file pone.0268013.s001.docx]
