# Supplementary material for: Early use of barbiturates is associated with increased mortality in traumatic brain injury patients from a propensity score-based analysis of a prospective cohort
Source: PLoS One. 2022 May 4;17(5):e0268013. doi: 10.1371/journal.pone.0268013 (PMC9067881; doi:10.1371/journal.pone.0268013)
Supplement: Attachment — Submitted filename: Pento.response.final.docx [file pone.0268013.s003.docx]

Maxime Léger

Anesthesiology and Critical Care Department,

Angers University Hospital,

4 rue Larrey - 49 933 Angers Cedex 9 – France

[mxmleger@gmail.com](mailto:mxmleger@gmail.com)

Twitter: @mxmleger

Academic Editor

Plos One

November 21, 2021

Dear editor,

I am pleased to submit an original work entitled “Early use of barbiturates is associated with increased mortality in traumatic brain injury patients—a propensity score-based analysis of a prospective cohort.” for consideration for publication in *Plos One.*

We submit our manuscript as a new submission as we were in a reviewing process in *Plos One* on our previous manuscript (PONE-D-20-37630). We missed the response time. However, we did take into consideration the comments made by the reviewers and we offer a deeply revised version of our manuscript. We hope that we have addressed all the concerns and believe that these modifications have improved the manuscript substantially.

We remain convinced that our publication is suitable for publication in *Plos One.* Traumatic brain injury constitutes a considerable portion of the global injury burden, with approximately sixty million individuals who suffer traumatic brain injury from all causes each year in the world. We were particularly interested in the early prescription of barbiturates for the treatment of traumatic brain injury complicated of intracranial hypertension, even if this treatment is indicated as third-line alternative in this context (Seattle International Severe Traumatic Brain Injury Consensus Conference), few data are available that assess its benefit/risk balanced.

Our study analyzing the outcomes of 383 head injury patients from a French prospective multicenter cohort found an association with increased intensive care unit mortality. In light of these results, it seems necessary to question the place of the early use of barbiturates in our therapeutic arsenal in patients with head trauma.

This paper has not been, nor will be, published in whole or in part by any other journal. We have no conflicts of interest to disclose. We have read and understood your journal’s policies, and we believe that neither the manuscript nor the study violates any of these. We have anonymized our dataset and can make it accessible in order to reproduce the analyses.

The authors receive no specific funding for this work.

Thank you for your consideration.

Sincerely,

Dr. Maxime Léger

Anesthesiology and Critical Care Department, Angers University Hospital, France.
