# Supplementary material for: Early use of barbiturates is associated with increased mortality in traumatic brain injury patients from a propensity score-based analysis of a prospective cohort
Source: PLoS One. 2022 May 4;17(5):e0268013. doi: 10.1371/journal.pone.0268013 (PMC9067881; doi:10.1371/journal.pone.0268013)
Supplement: Attachment — Submitted filename: response_reviewers.docx [file pone.0268013.s004.docx]

**Manuscript no.** PONE-D-20-37630R1

Title: Early use of barbiturates is associated with increased mortality in traumatic brain injury patients—a propensity score-based analysis of a prospective cohort.

Corresponding author:

Maxime Léger

Anaesthesiology and Critical Care Department,

Angers University Hospital,

4 rue Larrey - 49 933 Angers Cedex 9 – France

[mxmleger@gmail.com](mailto:mxmleger@gmail.com)

February 14, 2022

Dear Aurel Popa-Wagner,

I am pleased to submit on behalf of my co-authors this revised version of our manuscript entitled “Early use of barbiturates is associated with increased mortality in traumatic brain injury patients—a propensity score-based analysis of a prospective cohort.” for possible publication in *Plos One.*

We thank you and the reviewer for your careful reading of our manuscript. Please find bellow the point-to-point responses to the reviewer comments. We hope that we have addressed all the concerns and believe that these modifications have improved the manuscript substantially.

Thank you for your time and consideration.

Yours Sincerely,

Maxime Léger	14/02/2022

**Reviewer #2**

*The following concerns should be addressed before publication:*

*1. The aim of the study is evaluate the effect of barbiturate therapy in patients with TBI and intracranial hypertension on outcomes including ICU survival. Patients who received early barbiturate therapy did so because clinicians believed there was an indication for barbiturates (and control patients did not receive barbiturates as clinicians presumably did not think barbiturates were indicated). It is challenging to draw conclusions about the effect of barbiturate therapy on outcomes where all factors leading to a clinician decision to prescribe barbiturates cannot be controlled for. This significant limitation should be clearly outlined in the discussion.*

We thank reviewer 2 for highlighting this point which is major in our analysis. In order to consider this proposal, we have ended our discussion with the following sentences:

“Finally, it is a challenge to draw conclusions when all the factors leading the clinician to prescribe barbiturates cannot be controlled. Only a randomized controlled study may help to control these limits and to clarify the benefit of barbiturates in severe TBI patients.”

*2. The authors also report that the findings are not in agreement with the result of a Cochrane review of RCTs. The authors report this is not surprising, since barbiturates have been shown to impair cerebral oxygenation and may thus impair outcomes. This ignores the difference in study design and the authors should acknowledge the weaknesses of an observational design compared to RCTs.*

The reviewer is right to point out that our analysis from observational data differs from the Cochrane review of randomized trials.

Our goal was to follow the rules of causal inference in order to reasonably emulate a pseudo-randomized trial (via the use of propensity scores). We are aware of the limitations of our analysis.

In order to clarify this point for readers, we have completed the end of our discussion by emphasizing the importance of conducting a randomized trial (see previous point):

“Finally, it is a challenge to draw conclusions when all the factors leading the clinician to prescribe barbiturates cannot be controlled. Only a randomized controlled study may help to control these limits and to clarify the benefit of barbiturates in severe TBI patients.”

In addition, we also propose to complete the sentence in the discussion referring to the Cochrane review as follows:

“This finding is not in agreement with the result of the Cochrane review [9], and while all the precautions of causal inference have been considered, these findings emerged from an analysis of observational data (contrary to the Cochrane review which includes randomized trials).”

*3. Please be consistent in the use of TBI patients (at times TBI patients is used and at times, brain-injured patients is used).*

As proposed, we have revised the manuscript to be consistent on the following term "TBI patients".
